# Machine learning advances the integration of covariates in population pharmacokinetic models: Valproic acid as an example

**DOI:** 10.3389/fphar.2022.994665

**Published:** 2022-10-17

**Authors:** Xiuqing Zhu, Ming Zhang, Yuguan Wen, Dewei Shang

**Affiliations:** ^1^ Department of Pharmacy, The Affiliated Brain Hospital of Guangzhou Medical University, Guangzhou, China; ^2^ Guangdong Engineering Technology Research Center for Translational Medicine of Mental Disorders, Guangzhou, China

**Keywords:** machine learning, covariate, population pharmacokinetic, valproic acid, therapeutic drug monitoring, XGBoost, shap, Monte Carlo simulation

## Abstract

**Background and Aim:** Many studies associated with the combination of machine learning (ML) and pharmacometrics have appeared in recent years. ML can be used as an initial step for fast screening of covariates in population pharmacokinetic (popPK) models. The present study aimed to integrate covariates derived from different popPK models using ML.

**Methods:** Two published popPK models of valproic acid (VPA) in Chinese epileptic patients were used, where the population parameters were influenced by some covariates. Based on the covariates and a one-compartment model that describes the pharmacokinetics of VPA, a dataset was constructed using Monte Carlo simulation, to develop an XGBoost model to estimate the steady-state concentrations ( $C_{ss}$ ) of VPA. We utilized SHapley Additive exPlanation (SHAP) values to interpret the prediction model, and calculated estimates of VPA exposure in four assumed scenarios involving different combinations of *CYP2C19* genotypes and co-administered antiepileptic drugs. To develop an easy-to-use model in the clinic, we built a simplified model by using *CYP2C19* genotypes and some noninvasive clinical parameters, and omitting several features that were infrequently measured or whose clinically available values were inaccurate, and verified it on our independent external dataset.

**Results:** After data preprocessing, the finally generated combined dataset was divided into a derivation cohort and a validation cohort (8:2). The XGBoost model was developed in the derivation cohort and yielded excellent performance in the validation cohort with a mean absolute error of 2.4 mg/L, root-mean-squared error of 3.3 mg/L, mean relative error of 0%, and percentages within ± 20% of actual values of 98.85%. The SHAP analysis revealed that daily dose, time, CYP2C19*2 and/or *3 variants, albumin, body weight, single dose, and CYP2C19*1*1 genotype were the top seven confounding factors influencing the $C_{ss}$ of VPA. Under the simulated dosage regimen of 500 mg/bid, the VPA exposure in patients who had CYP2C19*2 and/or *3 variants and no carbamazepine, phenytoin, or phenobarbital treatment, was approximately 1.74-fold compared to those with CYP2C19*1/*1 genotype and co-administered carbamazepine + phenytoin + phenobarbital. The feasibility of the simplified model was fully illustrated by its performance in our external dataset.

**Conclusion:** This study highlighted the bridging role of ML in big data and pharmacometrics, by integrating covariates derived from different popPK models.

## 1 Introduction

Model-informed precision dosing (MIPD), an emerging, modern approach for individualizing drug therapy, involves various mathematical modeling methods (e.g., pharmacometrics) to integrate multidimensional patient-level data (Darwich et al., 2021). In particular, machine learning (ML), as a new promising data-driven tool in MIPD, has attracted considerable attention recently (Kluwe et al., 2021). For example, a previous study by us proved the feasibility of ML algorithms for predicting the dose-adjusted concentrations of lamotrigine for personalized dose adjustment (Zhu et al., 2021a). Although a lot of related work has been conducted to directly predict drug concentration or drug dose using ML-based strategies (Jovanović et al., 2015; Huang et al., 2021a; Zheng et al., 2021), the integration of model-informed and data-driven approaches is critical (Kluwe et al., 2021).

Fortunately, research collaborations among experts in different fields are advancing the integration of these approaches. Tang et al. (2021) reported a combined population pharmacokinetic (popPK) and ML approach, which had more accurate predictions of individual clearances of renally eliminated drugs in neonates and could be used to individualize the initial dosing regimen. Bououda et al. (2022) also suggested that ML could be used in combination with standard popPK approaches to increase confidence in the predictions of vancomycin exposure. Ogami et al. (2021) developed a model by applying artificial neural networks for predicting the time-series pharmacokinetics of cyclosporine A, which showed higher prediction accuracy than the conventional popPK model. Woillard et al. (2021a) developed an eXtreme gradient boosting (XGBoost) model allowing accurate estimation of the area under the curve (AUC) of tacrolimus based on only two or three concentrations with excellent performance, better than that of deterministic pharmacokinetic models with Bayesian estimation. However, the major limitation to developing such accurate ML models is the availability of large databases on drug concentration*-*time profiles, which can be solved by using simulation methods such as Monte Carlo (MC) simulation (Woillard et al., 2021b). MC simulation results in estimations of the possible outcomes by expanding the sample size, in light of probability distributions of the relevant parameters as inputs in a model (Zhu et al., 2021b). This technique has been used for popPK models to determine remedial dosing recommendations for non-adherent patients (Wang et al., 2020; Liu et al., 2021). Another study by Sibieude et al. (2021) applied ML as a fast initial covariate screening strategy and then utilized more traditional pharmacometrics approaches to build a final satisfying model to assess the clinical relevance of selected covariates and make predictions in different populations and scenarios. Thus, pharmacometrics can partner with ML to advance clinical data science by strongly decreasing computational costs for analyzing clinical datasets (Koch et al., 2020; Sibieude et al., 2021). Nevertheless, to the best of our knowledge, few studies have explored integrating covariates derived from different popPK models using ML. Our study, therefore, fills this gap.

Valproic acid (VPA) is a widely used drug for the treatment of bipolar disorder, particularly for acute mania, and multiple seizure types such as generalized tonic-clonic seizures (Hakami, 2021; Kishi et al., 2022). As a narrow therapeutic index drug, it is characterized by high pharmacokinetic variability (Johannessen and Johannessen, 2003). Various popPK models of VPA in Chinese patients have been constructed in recent years, to explore personalized VPA dosing and its variability patterns (Xu et al., 2018; Zang et al., 2022a). However, the covariates that influence the VPA pharmacokinetics varied between these models. Therefore, it is necessary to investigate the comprehensive impacts of these potential factors on VPA pharmacokinetics using our established XGBoost model.

The XGBoost algorithm, one of the best-known ensemble techniques, was originally developed by Chen and Guestrin (2016). It is based on the basic idea of boosting and serves as an extension to gradient boosted decision trees (GBDT), where the decision trees are built serially and each tree tries to minimize the error made by the previous one (Yaman and Subasi, 2019). Several innovations have been made to the XGBoost algorithm, including parallel tree boosting and approximate greedy search (Chen and Guestrin, 2016). Therefore, it can simultaneously reduce the model bias and variance (Cao et al., 2010). This state-of-the-art ML algorithm has been gradually applied to deal with predictions of therapeutic drug monitoring (TDM) values, drug dose, and drug exposure to specific medications (Huang et al., 2021a; Guo et al., 2021; Bououda et al., 2022). The details of the differences between the XGBoost and GBDT algorithms are given in the section titled “An introduction to XGBoost algorithm.”

In this study, our objective was to integrate covariates derived from different popPK models of VPA using the XGBoost algorithm, interpret our proposed model based on the SHapley Additive exPlanations (SHAP) analysis (Lundberg and Lee, 2017), and evaluate the combined effects across multiple covariates (i.e., *CYP2C19* genotypes and co-administered enzyme-inducing antiepileptic drugs) in terms of VPA exposure by assuming four scenarios. Furthermore, for easy clinical use, we built a simplified model by using only *CYP2C19* genotypes and some noninvasive clinical parameters, and omitting several features (similar to the practices in the ablation experiment) that were infrequently measured during TDM [e.g., albumin (ALB)], or whose clinically available values were inaccurate [e.g., blood sampling time (t)]. We evaluated this simplified model on our independent external dataset. An easy-to-use web application based on the simplified model was then designed as a real-time tool to support clinical decisions for MIPD.

## 2 Materials and methods

### 2.1 Data source and dataset construction

Generally, the predictability of different popPK models when extrapolated to other clinical centers might remain to be compared (Lv et al., 2021). An external validation study based on published VPA models by Zang et al. (2022b) suggested that the absence of children, Asian ethnicity, one-compartment models, and inclusion of the covariates body weight (BW) and VPA dosage, were the most important factors contributing to good performance in their Chinese dataset. This indicates that the selection of published popPK models of VPA is vital in our study, and priority may be given to these models that include the abovementioned factors. Moreover, glucuronidation and β-oxidation in the mitochondria are the major routes of VPA metabolism in humans (Ghodke-Puranik et al., 2013), and cytochrome P450 2C9 (CYP2C9) is the most significant cytochrome P450 (CYP) enzyme that mediates the oxidation of VPA considered a minor route of its metabolism (Ho et al., 2003; Ghodke-Puranik et al., 2013). Nevertheless, cytochrome P450 2C19 (CYP2C19) also participates in VPA metabolism (Hiemke et al., 2018; Song et al., 2022). Multiple studies reported that *CYP2C19* polymorphisms/genotypes significantly influenced the pharmacokinetic variability of VPA in Chinese Han subjects (Jiang et al., 2009; Guo et al., 2020; Wang et al., 2021). Given the limitations of the genetic test items in our hospital (no *CYP2C9* genotype testing), the reported references about the impact of *CYP2C19* polymorphisms on VPA, and the goal of validation of the simplified XGBoost model using our external dataset, we selected two previously published popPK models of VPA in Chinese epileptic patients for simulations [i.e., Model-A including the covariate *CYP2C19* genotypes (Guo et al., 2020) and Model-B including the covariates BW and daily dose of VPA (Daily Dose) (Lin et al., 2015)], both of which involved one-compartment models and Chinese epileptic patients aged 14 years and above. The detailed descriptions of the two studies are listed in Table 1.

**TABLE 1:** Descriptions of the two studies about Model-A and Model-B.

Items	Model-A (Guo et al., 2020)	Model-B (Lin et al., 2015)
Study design	A prospective study	A prospective study
Subjects	Chinese patients with seizures aged ≥18 years old in General Hospital of Taiyuan Iron and Steel (Group) Corporation (TISCO)	Chinese epileptic patients with normal liver and renal functions and 14 years of age or older in Huashan Hospital (Shanghai), Changzheng Hospital (Shanghai), Children’s Hospital (Shanghai), Tiantan Hospital (Beijing), and Brain Hospital (Nanjing)
Sample collection	Steady-state VPA serum concentration data were collected from January to December 2018	VPA serum samples at a steady state before the morning dose were collected between 1 October 1998, and 31 October 2003
Model description	One-compartment model	One-compartment model
Number of patients	60	199
Number of measurements	98	247
Age (years)	60 ± 11.8 (22–88)	26.6 ± 11.7 (14–66)
Gender (male/female)	44/16	114/85
Daily dose of VPA (mg)	500 (200–1,200)	884.5 ± 317.7 (250–1800)
VPA concentration (mg/L)	<150	61.9 ± 26.8 (3.2–140.3)
Formulation of VPA	Standard VPA dosing regimens (i.e., oral: 500 mg [immediate release tablets/solutions], twice per day; intravenous: 400 mg, twice per day)	VPA was prescribed 1–4 times a day and was administered orally in the forms of sustained-release tablets (Depakine, Sanofi-Aventis Pharmaceutical Ltd., Hangzhou, China) or conventional tablets (Hunan Xiangzhong Pharmaceutical Ltd., China)
Concomitant medications	Other medications that affect VPA concentrations were excluded (e.g., phenobarbital, carbamazepine, meropenem, imipenem, etc.)	Carbamazepine, phenytoin, phenobarbital, topiramate, and clonazepam

**FIGURE 1:**
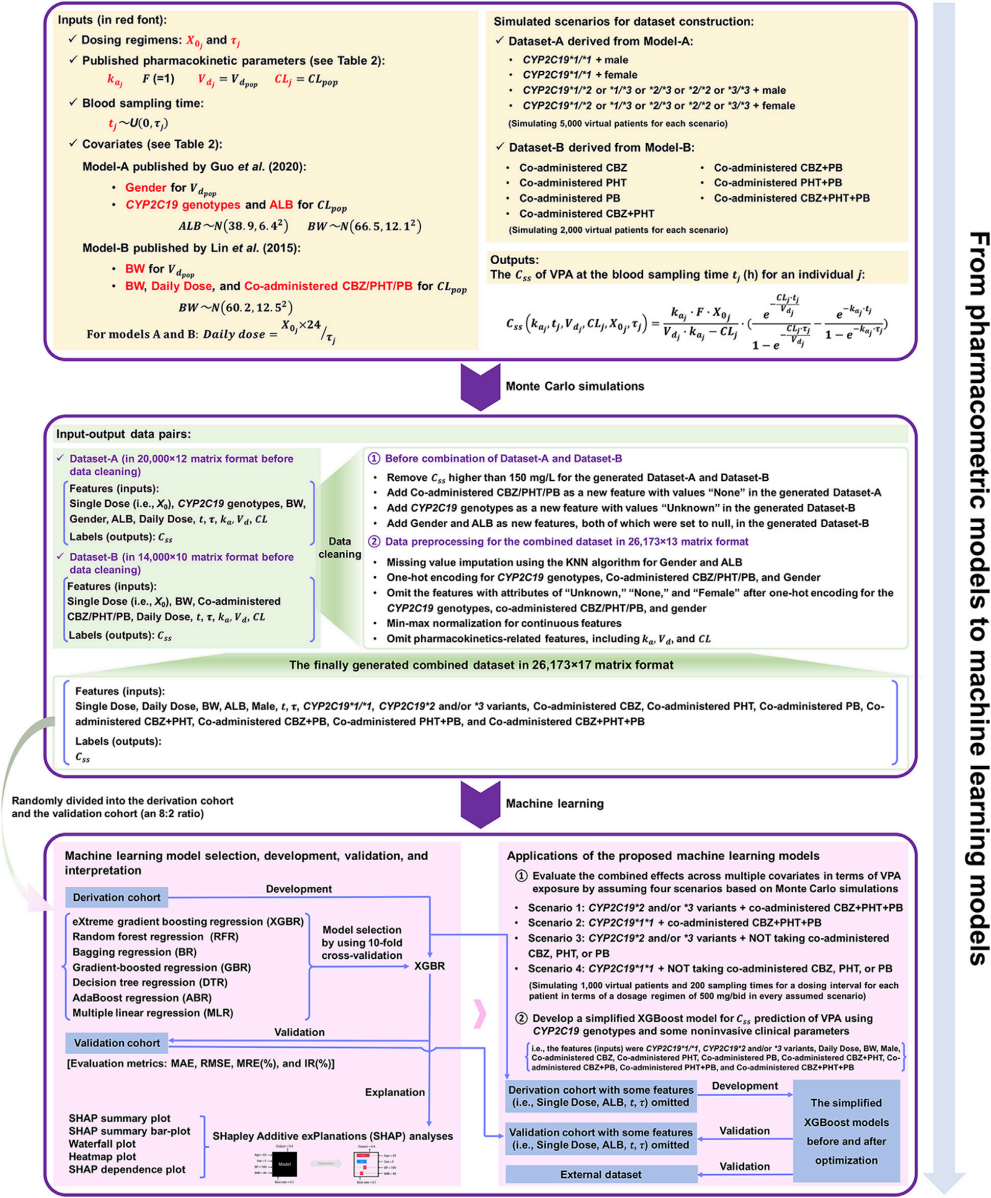
The workflow from pharmacometrics models to machine learning (ML) models mainly involves three parts: 1) data acquirement from published pharmacokinetic studies, 2) the construction of the combined dataset via Monte Carlo (MC) simulation and a series of data cleaning process, and 3) ML-based predictive modelling based on the finally generated combined dataset.

A general overview of our implementation of pharmacometric models to ML models in this study is shown in Figure 1. The population parameters, namely, the rate of absorption ( $k_{a}$ ), the apparent volume of distribution ( $V_{d}$ ), and the total serum clearance ( CL ), of Model-A and Model-B, were used to simulate the individual steady-state concentrations ( $C_{ss}$ ) of VPA, whose concentration-time profiles have been described by a one-compartment model, described as follows:

$$C_{ss}\left(k_{a_{j}},t_{j},V_{d_{j}},{CL}_{j},{X_{0}}_{j},\tau_{j}\right)=\frac{k_{a_{j}}∙F∙{X_{0}}_{j}}{V_{d_{j}}∙k_{a_{j}}-{CL}_{j}}∙\left(\frac{e^{-\frac{{CL}_{j}∙t_{j}}{V_{d_{j}}}}}{1-e^{-\frac{{CL}_{j}∙\tau_{j}}{V_{d_{j}}}}}-\frac{e^{-k_{a_{j}}∙t_{j}}}{1-e^{-k_{a_{j}}∙\tau_{j}}}\right)$$

where $C_{ss}\left(k_{a_{j}},t_{j},V_{d_{j}},{CL}_{j},{X_{0}}_{j},\tau_{j}\right)$ , $k_{a_{j}},$ $V_{d_{j}},\;{CL}_{j},$ ${X_{0}}_{j}$ , and $\tau_{j}$ are the $C_{ss}$ of VPA (mg/L) at the blood sampling time $t_{j}$ (h), the $k_{a}$ (h^−1^), the $V_{d}$ (L), the CL (L/h), a single dose (mg), and the dosing interval (h) for an individual j , respectively, F is the absolute bioavailability (%).

To determine a clear relationship between the features and the simulated outcomes without noise, $V_{d_{j}}$ and ${CL}_{j}$ are calculated using the following formulas without considering their inter-individual random effects (Mould and Upton, 2013):

$$V_{d_{j}}=V_{d_{pop}}$$

$${CL}_{j}={CL}_{pop}$$

where $V_{d_{pop}}$ and ${CL}_{pop}$ represent the typical population values of $V_{d}$ and CL , respectively.

The parameter $k_{a}$ is fixed at 2.38 h^−1^ and 1.90 h^−1^ in Model-A and Model-B, respectively; that is to say, $k_{a_{j}}$ equals $k_{a}$ . F is assumed to be one because the absolute systemic availability of VPA was found to be complete for all commonly used formulations (Gugler and von Unruh, 1980; Romoli et al., 2019). For Model-A, the covariates acting on $V_{d_{pop}}$ included gender, those acting on ${CL}_{pop}$ included *CYP2C19* genotypes and ALB, while the covariates included in Model-B were BW, which influences both $V_{d_{pop}}$ and ${CL}_{pop}$ , the Daily Dose, and cotherapy with enzyme-inducing antiepileptic drugs [including carbamazepine (CBZ), phenytoin (PHT), and phenobarbital (PB)] that influence ${CL}_{pop}$ . The related parameters in these models for the dataset simulation process are summarized in Table 2.

**TABLE 2:** Related parameters in the Model-A and Model-B for the dataset simulation process (in accordance with the original articles).

Models	Pharmacokinetic parameters			Covariates	
	$k_{a}$ (h^−1^)	$V_{d_{pop}}$ (L)	${CL}_{pop}$ (L/h)	BW (kg)	ALB (g/L)
Model-A Guo et al. (2020)	2.38	22.15 (if gender = female)	$0.64\times {\left(ALB/38.7\right)}^{-1.06}$ (if CYP2C19*1/*1)	66.5 ± 12.1	38.9 ± 6.4
		$22.15\times e^{0.78}$ (if gender = male)	$0.64\times {\left(ALB/38.7\right)}^{-1.06}\times e^{-0.45}$ (if CYP2C19*1/*2 or *1/*3 or *2/*3 or *2/*2 or *3/*3)		
Model-B Lin et al. (2015)	1.90	0.14×BW	$0.1\times {\left(BW/60\right)}^{0.7}\times {Daily\_Dose}^{0.2}\times 1.36\;\left(if\;cotherapy\;with\;CBZ\right)\times 1.25\;\left(if\;cotherapy\;with\;PHT\right)\times 1.11\;\left(if\;cotherapy\;with\;PB\right)$	60.2 ± 12.5	Not available

Note: Model-A had excluded other drugs that affect VPA concentrations (e.g., CBZ, PHT, or PB).

The constructed dataset combined two simulated datasets, i.e., Dataset-A and Dataset-B, derived from Model-A and Model-B, respectively. For Dataset-A, four scenarios (i.e., CYP2C19*1/*1 + male, CYP2C19*1/*1 + female, CYP2C19*1/*2 or *1/*3 or *2/*3 or *2/*2 or *3/*3 + male, and CYP2C19*1/*2 or *1/*3 or *2/*3 or *2/*2 or *3/*3 + female) were considered for simulating overall 20,000 virtual patients (in equal proportion, namely, simulating 5,000 virtual patients for each scenario). For each scenario, BW and ALB were simulated using normal distributions with mean ± standard deviation (SD) of (66.5 ± 12.1) kg and (38.9 ± 6.4) g/L, respectively, obtained from Model-A (see Table 2). For Dataset-B, a total of seven scenarios for different types of concomitant medication were simulated, including combinations with CBZ, PHT, PB, CBZ + PHT, CBZ + PB, PHT + PB, and CBZ + PHT + PB, and for each type, 2,000 virtual patients were generated, whose BW (kg) followed a normal distribution with 60.2 mean and an SD of 12.5, taken from Model-B (see Table 2). Dosing regimens were presumed to be the same in both models, as follows:

$${X_{0}}_{j}\in \left\{125,\;250,\;300,\;350,\;400,\;450,\;500,\;550,\;600,\;650,\;700,\;750,\;800,\;850,\;900\right\}(\mathrm{mg})$$

$$\tau_{j}\in \{6,\;8,\;12,\;24\}\left(h\right)$$

where ${X_{0}}_{j}$ and $\tau_{j}$ were sampled at random with the probability equal to 1/15 and 1/4, respectively. $t_{j}$ was assumed to have a uniform distribution of values between 0 and $\tau_{j}$ h.

Subsequently, MC simulations resulted in 20,000 and 14,000 individual values of $C_{ss}$ for Dataset-A and Dataset-B, respectively. Notably, types of concomitant medication (i.e., co-administered CBZ/PHT/PB) as a new feature, the values of which were “None,” was added in the generated Dataset-A because drugs that affect VPA concentrations had been excluded in Model-A; similarly, *CYP2C19* genotypes, as a new feature with values “Unknown,” were added in the generated Dataset-B owing to the unknown distributions of the values of this covariate (i.e., the proportions of the genotypes CYP2C19*1/*1, CYP2C19*1/*2, CYP2C19*1/*3, etc.). This was also not included in Model-B. However, gender and ALB, both of which were not covariates for Model-B, were set to null as new features in the generated Dataset-B due to their missing value imputation. To obtain less noise, filters were applied to both models to remove $C_{ss}$ higher than 150 mg/L to obtain a range of values compatible with observed data reported in the original articles (Woillard et al., 2021b), resulting in 14,509 and 11,664 $C_{ss}$ values retained in the finally generated Dataset-A and Dataset-B, respectively. Moreover, to ensure high-quality data containing as much useful information as possible to facilitate the training and test of the ML models, for the combined dataset in 26,173 × 13 matrix format [i.e., 26,173 simulated input–output data pairs (Dataset-A: Dataset-B = 14,509: 11,664)], we used the k-nearest neighbor imputation for gender and ALB. Both had 44.57% (11,664/26,173 × 100%) missing data (Beretta and Santaniello, 2016). We used one-hot encoding for categorical variables (Lopez-Arevalo et al., 2020), and min-max normalization for continuous feature variables, and then omitted the features with attributes of “Unknown,” “None,” and “Female” after one-hot encoding for the *CYP2C19* genotypes, co-administered CBZ/PHT/PB, and gender (considering the increased dimensionality of the dataset and the issue of collinearity because one of the categories could be completely generated from the others). We also omitted the pharmacokinetics-related features that are not easily available in the clinic (including $k_{a}$ , $V_{d}$ , and CL ). The combined dataset was finally generated after data preprocessing, including 26,173 $C_{ss}$ values and 16 features (i.e., Single Dose, BW, ALB, t , τ , Daily Dose, CYP2C19*1/*1, CYP2C19*2 and/or *3 variants (i.e., CYP2C19*1/*2 or *1/*3 or *2/*2 or *2/*3 or *3/*3), Male, Co-administered CBZ, Co-administered PHT, Co-administered PB, Co-administered CBZ + PHT, Co-administered CBZ + PB, Co-administered PHT + PB, and Co-administered CBZ + PHT + PB). Among these 16 features, the values of the categorical variables were one (=yes) or zero (=no). The process of dataset construction is shown in Figure 1.

### 2.2 An introduction to the XGBoost algorithm

XGBoost, a gradient-boosting framework, was developed by a team led by Chen Tianqi at the University of Washington (Chen and Guestrin, 2016). It is an effective tool for tackling classification and regression problems using tabular data. Compared with GBDT, XGBoost uses a series of optimizations (Li et al., 2019; Chen et al., 2020). An important aspect is the application of an additional regularization term to the loss function to prevent overfitting. The objective function ( L ) of XGBoost is calculated as:

$$L=\underset{i}{\sum }l\left(\;{\hat{y}}_{i},y_{i}\right)+\underset{k}{\sum }\Omega \left(f_{k}\right)$$

where l is the loss function representing the error between the actual values ( $y_{i}$ ) and the predicted values ( ${\hat{y}}_{i}$ ), and $\Omega \left(f_{k}\right)$ is the regularized term, defined as:

$$\Omega \left(f_{k}\right)=\gamma T+\frac{1}{2}\lambda {\left\|\omega \right\|}^{2}$$

where Τ and ω represent the number of leaves in the tree and the corresponding weight of different leaves of each tree, respectively, and γ and λ are the regularized parameters that penalize Τ and ω , respectively.

Moreover, the second-order Taylor expansion of L can more efficiently fit the error. For the *t*-th iteration, *L* ^*(t)*^ is:

$$L^{\left(t\right)}≃\underset{i}{\sum }\left[l\left(y_{i},{\hat{y}}_{i}^{\left(t-1\right)}+g_{i}f_{t}\left(x_{i}\right)+\frac{1}{2}h_{i}f_{t}^{2}\left(x_{i}\right)\right)\right]+\Omega \left(f_{t}\right)$$

where $g_{i}=\partial_{{\hat{y}}_{i}^{\left(t-1\right)}}l\left(y_{i},{\hat{y}}_{i}^{\left(t-1\right)}\right)$ and $h_{i}=\partial_{{\hat{y}}_{i}^{\left(t-1\right)}}^{2}l\left(y_{i},{\hat{y}}_{i}^{\left(t-1\right)}\right)$ are the first- and second-order gradients, respectively.

Subsequently, other calculations were used to determine the optimal split node by using the information gain of L . This is another algorithmic innovation. Gain denotes the gain for each split of the tree. It is used to evaluate the candidate splits, and is given by:

$$Gain=\frac{1}{2}\left[\frac{{\left(\sum_{i\in I_{L}}g_{i}\right)}^{2}}{\sum_{i\in I_{L}}h_{i}+\lambda }+\frac{{\left(\sum_{i\in I_{R}}g_{i}\right)}^{2}}{\sum_{i\in I_{R}}h_{i}+\lambda }-\frac{{\left(\sum_{i\in I}g_{i}\right)}^{2}}{\sum_{i\in I}h_{i}+\lambda }\right]-\gamma$$

where $I_{L}$ and $I_{R}$ represent the instance sets of the left and right nodes after the split, respectively, and $I=I_{L}\cup I_{R}$ .

XGBoost has a multitude of hyperparameters. The optimal choice of the following key hyperparameters may yield the best performance by the model:

1) n_estimators: This represents the total number of trees. Too small or too large a value of *n_estimators* may lead to underfitting or overfitting, respectively.
2) max_depth: It is the maximum depth of the tree. Increasing *max_depth* will make the model more complex and lends it a stronger fitting ability. However, a large value is likely to cause it to overfit the data.
3) min_child_weight: It represents the minimum number of samples that a node can represent in order to be split further. We can increase this value to reduce overfitting.
4) gamma: It is a regularization parameter that denotes the minimum reduction in loss at every split. The larger *gamma* is, the more conservative the algorithm is, the smaller is the number of leaves that the tree has, and therefore, the lower is the complexity of the model.
5) colsample_bytree: It denotes the subsample ratio of columns (i.e., the rate of feature sampling) when constructing each tree, and controls overfitting.
6) subsample: It is the subsample ratio of the training instances. Increasing this value makes the algorithm more conservative and the model more likely to underfit.
7) learning_rate: It is the shrinkage in step size used in updates to prevent overfitting. Reducing the weight of each step makes the model more robust.

### 2.3 Model development and evaluation

The finally generated combined dataset in 26,173 × 17 matrix format was randomly divided into two parts, the derivation cohort for model selection and the development of the XGBoost model, and the validation cohort for its evaluation (an 8: 2 ratio). Before using the XGBoost algorithm, 10-fold cross-validation was applied to the derivation cohort to assess the performance of the XGBoost model, and other tree-based and non-tree-based models, including random forest regression (RFR), bagging regression (BR), gradient-boosted regression (GBR), decision tree regression (DTR), AdaBoost regression (ABR), and multiple linear regression (MLR). We used their default settings for the hyperparameters.

K-fold cross-validation involves 1) splitting the derivation cohort into K folds, 2) starting by using K-1 folds as the training set and the remaining one fold as the test set, 3) training the model on the training set and testing it on the test set, 4) saving the test score, 5) repeating steps 2–4 for K iterations, and 6) comparing the performance of the models by using the average cross-validation score [mean absolute error (MAE), used as the evaluation metric in this study] in the test sets across all K folds (Kalagotla et al., 2021).

The metrics used to evaluate the performance of the developed XGBoost model on the validation cohort were the MAE, root-mean-squared error (RMSE), mean relative error (MRE), and ideal rate (IR, i.e., percentages within ±20% of actual values), defined as follows:

$$MAE=\frac{\sum_{i=1}^{N}\left({{\hat{y}}_{i}-y}_{i}\right)}{N}$$

$$RMSE=\sqrt{\frac{\sum_{i=1}^{N}{\left({{\hat{y}}_{i}-y}_{i}\right)}^{2}}{N}}$$

$$MRE\left(\%\right)=\frac{\sum_{i=1}^{N}\left({{\hat{y}}_{i}-y}_{i}\right)/y_{i}}{N}\times 100\%$$

$$IR\left(\%\right)=\frac{N_{predicted\;values\;within\;\pm 20\%\;of\;actual\;vaules}}{N_{total\;actual\;vaules}}\times 100\%$$

where ${\hat{y}}_{i}$ and $y_{i}$ denote the predicted and actual values, respectively.

### 2.4 Model interpretation

The SHAP analysis was utilized to provide interpretability to the proposed XGBoost model, which is generally criticized as a ‘black-box’ model due to its complexity. The main advantages of SHAP inspired by cooperative game theory (Štrumbelj and Kononenko, 2014), are that it is model agnostic, easy to use, and straightforward to interpret the feature contributions at global and local levels, as well as the interactions among these features (Li et al., 2020). The contribution of each feature on the model output associated with each predicted sample is allocated according to their marginal contribution (Shapley, 1953), and can be determined by the Shapley value, defined via the following formula (Yang et al., 2021):

$$\phi_{i}\left(\nu \right)=\underset{S\subseteq \left\{1,\ldots ,p\right\}\setminus \left\{i\right\}}{\sum }\frac{\left|S\right|!\left(p-\left|S\right|-1\right)!}{p!}\left(\nu \left(S\cup \left\{i\right\}\right)-\nu \left(S\right)\right)$$

where $\phi_{i}\left(\nu \right)$ is the contribution of feature i , p is the number of features, S is a subset of the features used in the model, and ν(S∪{i})−ν(S) represents the influence of feature i on the improvement of the result (i.e., marginal contribution).

### 2.5 Applications of the integration of pharmacometrics and ML models

#### 2.5.1 Impacts of the integrated covariates on VPA exposure

To assess the comprehensive impacts of different popPK models-derived covariates–*CYP2C19* genotypes and co-administered enzyme-inducing antiepileptic drugs–on VPA exposure, we used MC simulations to simulate 1,000 virtual patients and 200 sampling times for a dosing interval (i.e., t , uniformly distributed between 0 and 12 h) for each patient in terms of a dosage regimen of 500 mg/bid in every assumed scenario. A total of 16 predictors [Single Dose (set to 500 mg), BW, ALB, t , τ (set to 12 h), Daily Dose (set to 1,000 mg), CYP2C19*1/*1, CYP2C19*2 and/or *3 variants, Male, Co-administered CBZ, Co-administered PHT, Co-administered PB, Co-administered CBZ + PHT, Co-administered CBZ + PB, Co-administered PHT + PB, and Co-administered CBZ + PHT + PB] were simulated based on the proposed XGBoost model. Among them, the BW and ALB were simulated as normal distributions, with mean ± SD described in the finally generated combined dataset (see Table 3), and the male and female patients were simulated with equal probabilities (i.e., the probability of Male = 1 was 0.5).

**TABLE 3:** Simulated patient characteristics in the finally generated combined dataset (N = 26,173).

Continuous data	Value [(mean ± SD) or median (min–max)]	Categorical data	Distribution [n (%)]
$C_{ss}$ (mg/L)	73.7 ± 37.2	Male	7,237 (27.65%)
BW (kg)	64.3 ± 12.4	CYP2C19*1/*1	8,358 (31.93%)
ALB (g/L)	37.9 ± 5.6	CYP2C19*2 and/or *3 variants	6,151 (23.50%)
Daily Dose (mg)	900 (125–3,600)	Co-administered CBZ	1,636 (6.25%)
Single Dose (mg)	450 (125–900)	Co-administered PHT	1,529 (5.84%)
t (h)	5.57 (0–24)	Co-administered PB	1,421 (5.43%)
τ (h)	12 (6–24)	Co-administered CBZ + PHT	1,854 (7.08%)
		Co-administered CBZ + PB	1,735 (6.63%)
		Co-administered PHT + PB	1,652 (6.31%)
		Co-administered CBZ + PHT + PB	1,837 (7.02%)

Note: Css denotes the steady-state concentrations of VPA, t denotes the blood sampling time, and τ denotes the dosing interval.

A total of four scenarios were considered:

Scenario 1: Patients with CYP2C19*2 and/or *3 variants (feature value = 1) and taking co-administered CBZ + PHT + PB (feature value = 1).

Scenario 2: Patients with CYP2C19*1*1 genotype (feature value = 1) and taking co-administered CBZ + PHT + PB (feature value = 1).

Scenario 3: Patients with CYP2C19*2 and/or *3 variants (feature value = 1) and *NOT* taking co-administered CBZ, PHT, or PB (feature values of all co-administered drug predictors = 0).

Scenario 4: Patients with CYP2C19*1*1 genotype (feature value = 1) and *NOT* taking co-administered CBZ, PHT, or PB (feature values of all co-administered drug predictors = 0).

All predictors except for t were considered to be constant for each virtual patient. Therefore, these static values were replicated across t , resulting in tabular data in which each scenario had 1,000 × 200 samples for predictions of $C_{ss}$ by using our proposed XGBoost model. The concentration-time profiles were then plotted for all scenarios using the two visualization libraries matplotlib and seaborn. The VPA exposures [i.e., ${AUC}_{0\to 12h}$ (mg・h/L)] in the aforementioned scenarios were obtained using the trapezoidal rule by dividing the curve’s total area into small trapezoids rather than dividing it into small rectangles (Woillard et al., 2021b), and the average $C_{ss}$ ( ${\bar{C}}_{ss}$ ) (mg/L) was calculated as follows:

$${\bar{C}}_{ss}={AUC}_{0\to 12h}/12$$

Both ${AUC}_{0\to 12h}$ and ${\bar{C}}_{ss}$ were calculated in Python by using the numpy package.

#### 2.5.2 Model simplification to develop an easy-to-use MIPD tool

In clinical practice, a balance needs to be struck between the performance of the ML model and its ease of use. The ideal ML models are those that have as few predictors as possible (and perhaps should be easily available in the clinic) while delivering high performance. In this study, we aimed to build a simplified XGBoost model to develop an easy-to-use MIPD tool. Considering that the values of some predictors were missing owing to infrequent measurements during TDM (e.g., ALB) or were inaccurate clinical data (e.g., inappropriate sampling time in the TDM practice and irregular single doses or dosing intervals in the prescriptions) (Jakobsen et al., 2017; Firman et al., 2021), we built a simplified model by omitting these types of features (i.e., Single Dose, ALB, t , τ ) in the final, combined dataset. We developed an easy-to-use model in the clinic by using only *CYP2C19* genotypes and some noninvasive clinical parameters as predictors, and observed the influence of the omitted predictors on the performance of the proposed XGBoost model. Finally, we optimized the hyperparameters via the sklearn’s own grid search approach using the evaluation metric of MAE and tenfold cross-validation (Radzi et al., 2021), and verified this simplified model after optimization in our independent external dataset, which consisted of 105 input-output data pairs retrospectively collected from our routine TDM practice according to guidelines of the Ethics Committee of the Affiliated Brain Hospital of Guangzhou Medical University approval ([2021] NO.027). The inputs to the external dataset were the same as those of the finally generated combined dataset with Single Dose, ALB, t , and τ omitted. They consisted of CYP2C19*1/*1, CYP2C19*2 and/or *3 variants, Daily Dose, BW, Male, Co-administered CBZ, Co-administered PHT, Co-administered PB, Co-administered CBZ + PHT, Co-administered CBZ + PB, Co-administered PHT + PB, and Co-administered CBZ + PHT + *p*B. The external dataset is described in Table 4. We designed an easy-to-use web application based on the simplified optimum XGBoost model to realize real-time estimations of values of $C_{ss}$ of the VPA by automatically crawling information on the model inputs from the electronic health record (EHR) system.

**TABLE 4:** Descriptions of our external dataset.

Items	Value
Number of patients	56
Total number of measured steady-state VPA concentrations	105
Average TDM measurements per patient	1.88
Age (years, mean ± SD)	34.48 ± 13.10
BW (kg, mean ± SD)	63.82 ± 11.48
Gender	
Male	42
Female	14
The number of patients with the *CYP2C19* genotype of	
CYP2C19*1/*1	22
CYP2C19*1/*2	26
CYP2C19*1/*3	4
CYP2C19*2/*2	1
CYP2C19*2/*3	3
Daily dose [mg, median (min–max)]	1,000 (250–2000)
$C_{ss}$ (mg/L)	87.3 ± 22.8

Note: All patients did not take co-administered CBZ/PHT/PB.

### 2.6 Implementation

All the analyses were performed in Python using the Jupyter notebook. Libraries sklearn, XGBoost, pandas, numpy, scipy, matplotlib, seaborn, palettable, and shap, were used for implementation.

## 3 Results

### 3.1 Simulation and data

**FIGURE 2:**
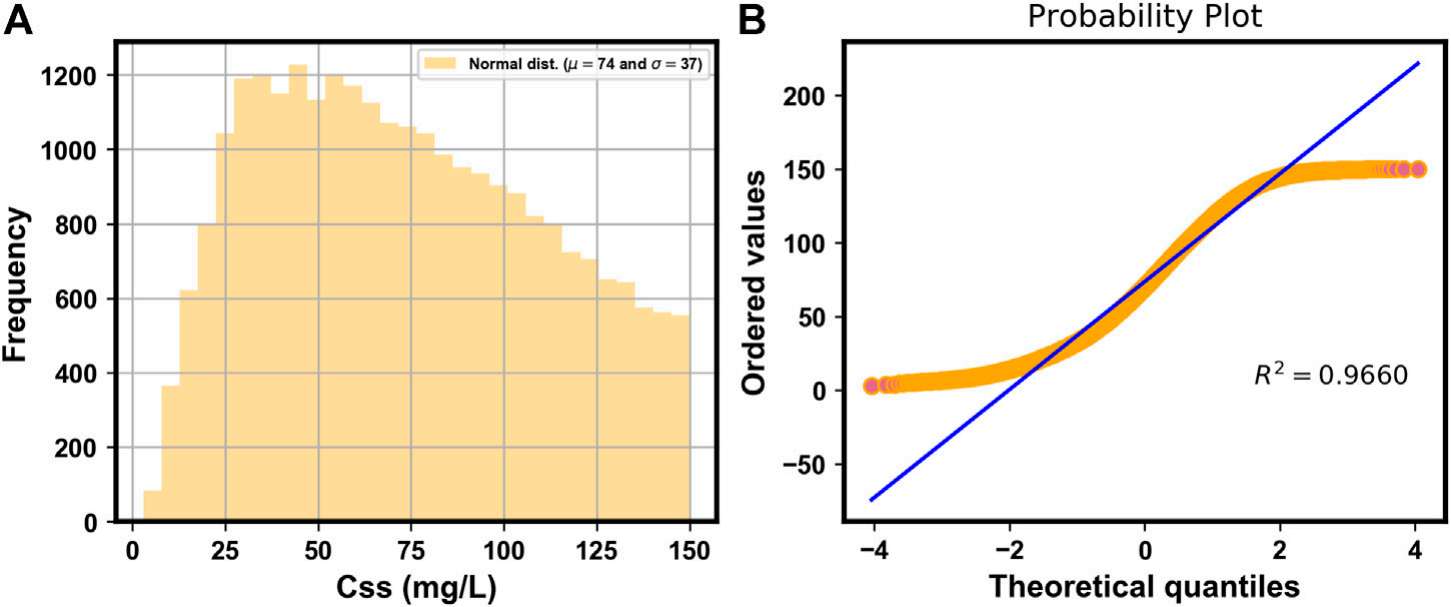
Histogram **(A)** and probability plot **(B)** of the simulated $C_{ss}$ of VPA.

**FIGURE 3:**
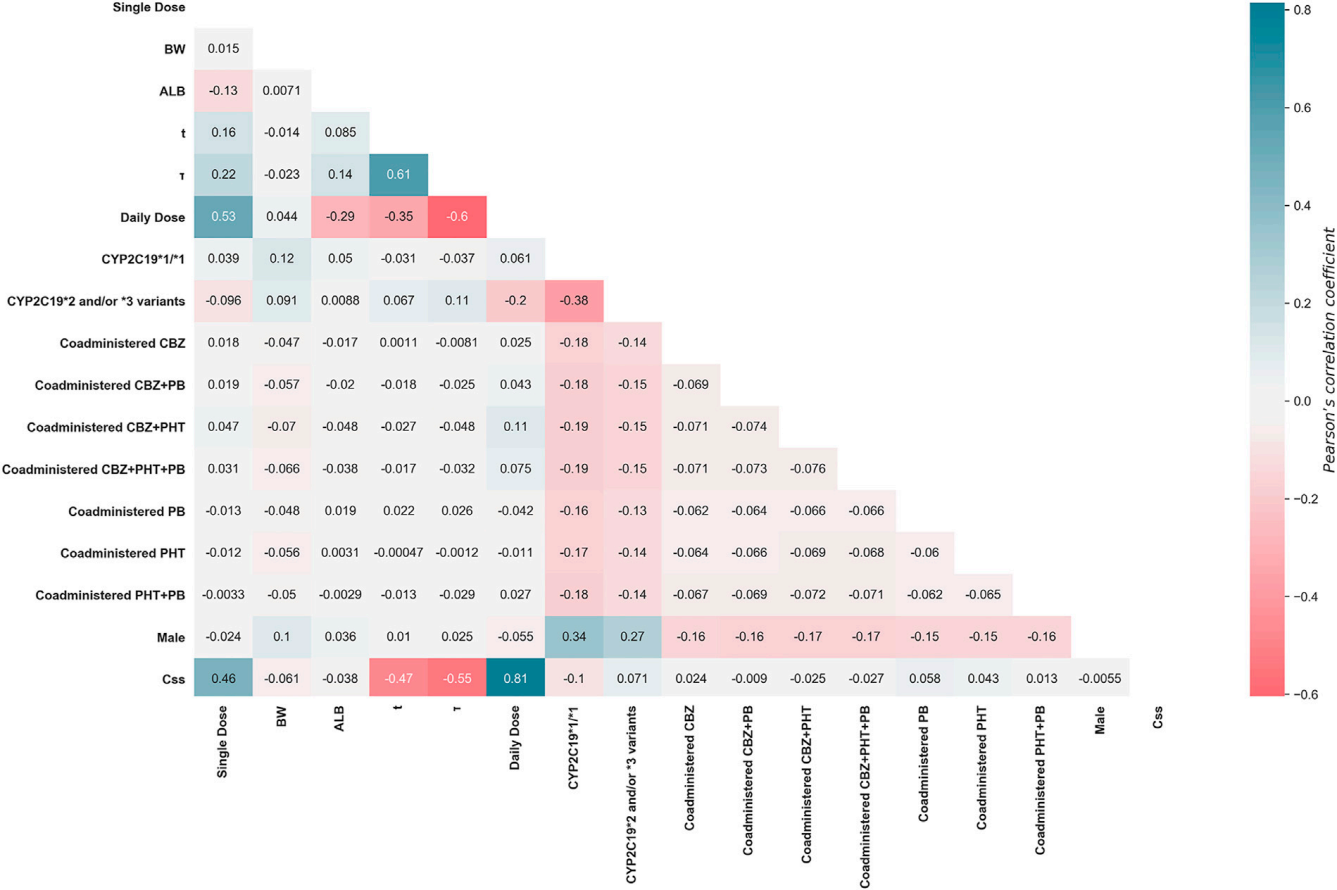
Heat map of Pearson’s correlations between $C_{ss}$ of VPA and features.

Figure 2A shows the histogram of the simulated $C_{ss}$ of VPA, whose probability plot indicated a normal distribution (*R* ^2^ = 0.9660) (Figure 2B). Figure 3 shows a heat map of the Pearson’s correlation coefficients between the $C_{ss}$ of VPA and features, indicating that “Daily Dose” and “ τ ” were the most important positive and negative predictors correlated with $C_{ss}$ , respectively, and no obvious multi-collinear relationships were observed between the features. The characteristics of the simulated patients in the finally generated combined dataset are shown in Table 3.

### 3.2 XGBoost model

Table 5 shows the overall comparison of the regression models in the derivation cohort. The lowest average MAE value of the XGBoost model in the test sets indicated that it was superior to the other tree-based and non-tree-based models considered. As is presented in Table 6, the proposed XGBoost model delivered excellent performance on the validation cohort, illustrated by an MAE of 2.4 mg/L, RMSE of 3.3 mg/L, MRE of 0%, and IR of 98.85%, respectively.

**TABLE 5:** The mean absolute error (MAE) at 95% confidence interval (CI) for the prediction of the value of $C_{ss}$ of VPA in the derivation cohort for the XGBoost and other regression models.

Models	Training set		Test set	
	MAE (mg/L)	(+/-) 95% CI of MAE (mg/L)	MAE (mg/L)	(+/-) 95% CI of MAE (mg/L)
XGBR	1.7	0.1	2.5	0.1
RFR	1.2	0	3.1	0.2
BR	1.5	0	3.5	0.2
DTR	0	0	5.1	0.3
GBR	6.0	0.1	6.2	0.2
MLR	10.2	0	10.2	0.4
ABR	14.1	0.3	14.2	0.7

**TABLE 6:** Comparisons of the performance of the proposed models on the validation cohort and the independent external dataset.

Datasets	Models	Descriptions of models		Evaluation metrics			
		Selected features	Hyperparameters	MAE (mg/L)	RMSE (mg/L)	MRE (%)	IR (%)
Validation cohort (N = 5,235)	XGBoost model	Single Dose, BW, ALB, t , τ , Daily Dose, CYP2C19*1/*1, CYP2C19*2 and/or *3 variants, Male, Co-administered CBZ, Co-administered PHT, Co-administered PB, Co-administered CBZ + PHT, Co-administered CBZ + PB, Co-administered PHT + PB, and Co-administered CBZ + PHT + PB	Default settings	2.4	3.3	0	98.85
	Simplified XGBoost model	BW, Daily Dose, CYP2C19*1/*1, CYP2C19*2 and/or *3 variants, Male, Co-administered CBZ, Co-administered PHT, Co-administered PB, Co-administered CBZ + PHT, Co-administered CBZ + PB, Co-administered PHT + PB, and Co-administered CBZ + PHT + PB	Default settings	11.2	14.7	5	68.00
	Simplified XGBoost model after optimization	BW, Daily Dose, CYP2C19*1/*1, CYP2C19*2 and/or *3 variants, Male, Co-administered CBZ, Co-administered PHT, Co-administered PB, Co-administered CBZ + PHT, Co-administered CBZ + PB, Co-administered PHT + PB, and Co-administered CBZ + PHT + PB	n_estimators: 20, max_depth: 6, min_child_weight: 5, gamma: 0, colsample_bytree: 1.0, subsample: 1.0, learning_rate: 0.3	11.0	14.4	5	69.11
External dataset (N = 105)	Simplified XGBoost model after optimization	BW, Daily Dose, CYP2C19*1/*1, CYP2C19*2 and/or *3 variants, Male, Co-administered CBZ, Co-administered PHT, Co-administered PB, Co-administered CBZ + PHT, Co-administered CBZ + PB, Co-administered PHT + PB, and Co-administered CBZ + PHT + PB	n_estimators: 20, max_depth: 6, min_child_weight: 5, gamma: 0, colsample_bytree: 1.0, subsample: 1.0, learning_rate: 0.3	16.5	20.1	13	60.00

### 3.3 SHAP analysis

**FIGURE 4:**
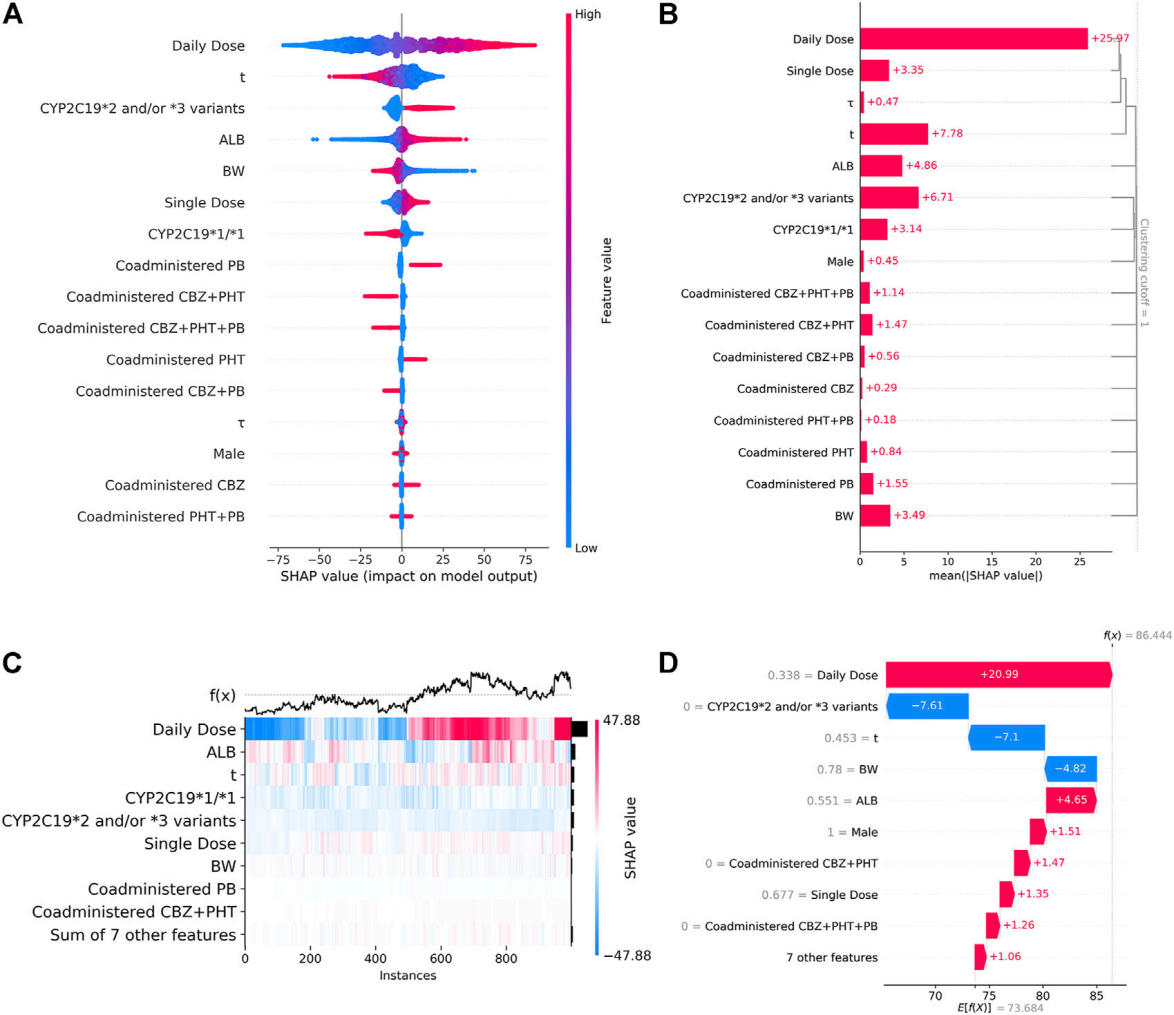
**(A)**. SHAP summary plot. From it, we can get an initial sense of the relationship between the value of a certain feature and its impact on prediction. Each point represents an instance and a Shapley value for a feature. Its position on this plot is determined by the feature on the *y*-axis (ordered by feature importance) and the Shapley value on the *x*-axis, while its color is determined by the value of the feature. A higher SHAP value corresponds to a higher $C_{ss}$ of VPA, and *vice versa*. **(B)** SHAP bar plot obtained by using feature clustering, from which we can simultaneously visualize the structure of the clustering and the importance of the features. The numbers on the histograms represent the mean (|SHAP value|) of a feature. SHAP analysis can also explain individual predictions, illustrated by **(C–D)**. **(C)** SHAP heat map, with the top 1,000 instances on the *x*-axis and the model inputs on the *y*-axis. The SHAP values encoded on a color scale. The model outputs are shown above the heatmap matrix and centered around the dotted gray baseline. The global importance of each feature is shown as a black bar plot on the right-hand side of the plot. **(D)** Waterfall plot that explains a single prediction of the sample randomly selected from the 1,000 instances by visualizing how to obtain the final prediction with the SHAP values of each feature. The bottom of the plot starts as expected, and then each row shows how the positive (red) or negative (blue) contribution of each feature moves the value from the expected output of the model, under the background distribution of the dataset, to its final prediction. The value of each feature for this sample appears in gray text before the feature name.

**FIGURE 5:**
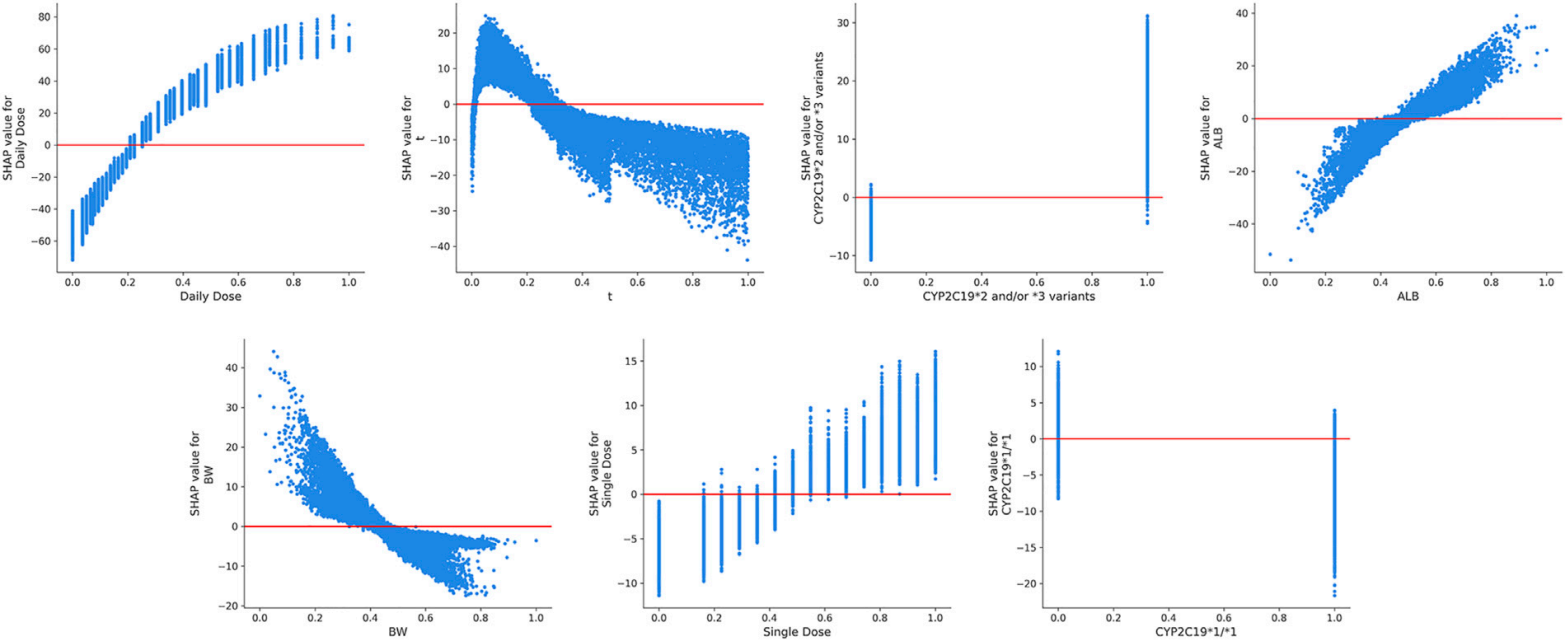
The SHAP dependence plots of features that ranked higher according to their importance ranking. From the scatter plots, we can see the exact form of the relationships between a single feature and the predictions made by the model.

Figure 4A shows the SHAP summary plot that orders all predictors according to their feature importance to detect the features which have high contributions to the $C_{ss}$ of VPA. Among these features, Daily Dose was ranked first, followed by t , CYP2C19*2 and/or *3 variants, ALB, BW, Single Dose, and CYP2C19*1/*1. Moreover, higher SHAP values of a feature indicated higher $C_{ss}$ of VPA, and *vice versa*. The colored dots determined the direction of influence, i.e., the higher the input value of a feature, the higher the $C_{ss}$ of VPA, when red dots were in the positive range of SHAP values. Likewise, Figure 4B shows the hierarchical feature clustering of the SHAP bar plot that sorts the feature importance values of each cluster and subcluster to show the most important features at the top. The global importance of the predictors was calculated according to the mean absolute SHAP values [mean (|SHAP value|)] of each feature over all instances (rows) of the finally generated combined dataset. SHAP can also explain individual predictions. Figure 4C shows the SHAP heat map of the top 1,000 instances extracted from the dataset. It ordered samples by using supervised clustering, and this resulted in samples that had the same model outputs, for the same reason for which they were grouped together. Figure 4D shows the applicability of the proposed XGBoost model on a single sample randomly selected from these 1,000 instances, where the highest contribution to the $C_{ss}$ of VPA is the Daily Dose (feature value = 0.338) and CYP2C19*2 and/or *3 variants (feature value = 0), and was generally not in agreement with the results of the global interpretations of the SHAP summary plot analysis. It indicated the potential difference in the rankings of the contributions of the features at the individual level. The SHAP dependence plots of the top seven key features are displayed in Figure 5, to show how a feature affected the $C_{ss}$ of VPA. Nonlinear associations between features (e.g., t ) and the $C_{ss}$ of VPA were observed. The results showed that higher Daily/Single Dose and ALB, lower BW, and CYP2C19*2 and/or *3 variants, were related to higher $C_{ss}$ of VPA.

### 3.4 Impacts of covariates on VPA exposure

**FIGURE 6:**
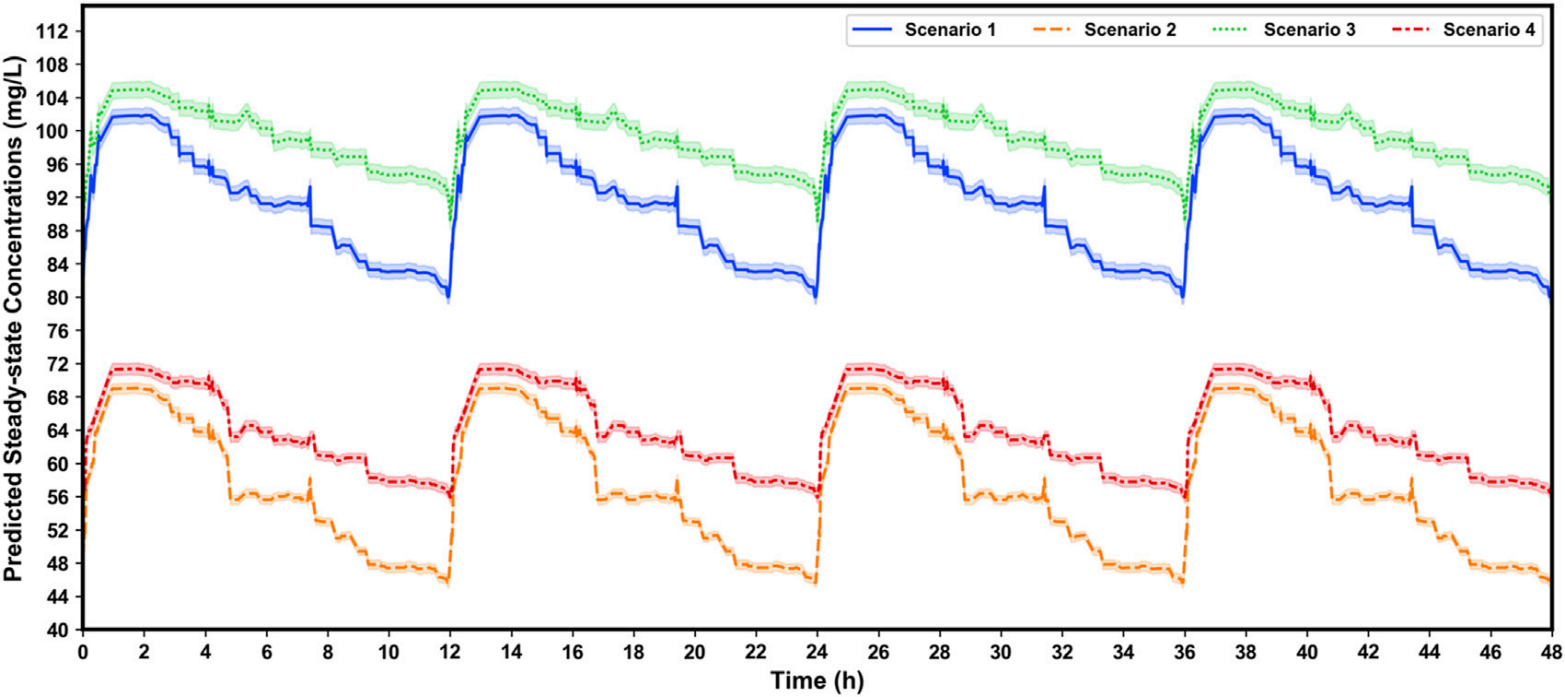
Simulated $C_{ss}$ of VPA plotted by using four dosing intervals at the dosage regimen of 500 mg/bid in different scenarios based on the proposed XGBoost model. The numbers of virtual patients at each time point is 1,000. The blue, orange, green, and red line denotes scenario 1 (patients with CYP2C19*2 and/or *3 variants, and taking co-administered CBZ + PHT + PB), scenario 2 (patients with CYP2C19*1*1 genotype, and taking co-administered CBZ + PHT + PB), scenario 3 (patients with CYP2C19*2 and/or *3 variants, and *not* taking co-administered CBZ, PHT, or PB), and scenario 4 (patients with CYP2C19*1*1 genotype, and *not* taking co-administered CBZ, PHT, or PB), respectively. The shaded area represents the 95% confidence interval.

Figure 6 shows the comprehensive impacts of *CYP2C19* genotypes and co-administered enzyme-inducing antiepileptic drugs on the $C_{ss}$ of VPA under the dosage regimen of 500 mg/bid, by simulating four scenarios using the XGBoost model. The simulated ${AUC}_{0\to 12h}$ values at a steady-state calculated by the trapezoidal rule and the corresponding ${\bar{C}}_{ss}$ values are listed in Table 7. Our results showed that patients who had the CYP2C19*2 and/or *3 variants and did not receive CBZ, PHT, or PB, had more VPA exposure [ ${AUC}_{0\to 12h}$ : (1,187.5 ± 183.5) *versus* (683.4 ± 103.7) mg·h/L, approximately 1.74-fold] and more ${\bar{C}}_{ss}$ [(99.0 ± 15.3) *versus* (56.9 ± 8.6) mg/L] than those of individuals with CYP2C19*1/*1 genotype and co-administered CBZ + PHT + PB.

**TABLE 7:** Simulated steady-state area under the curve from time zero to 12 h ( ${AUC}_{0\to 12h})$ and the corresponding average $C_{ss}$ ( ${\bar{C}}_{ss}$ ) values of VPA under the dosage regimen of 500 mg/bid in terms of four different scenarios based on the XGBoost model.

Scenarios	${AUC}_{0\to 12h}$ (mg·h/L)	${\bar{C}}_{ss}$ (mg/L)
Scenario 1	1,093.3 ± 170.2	91.1 ± 14.2
Scenario 2	683.4 ± 103.7	56.9 ± 8.6
Scenario 3	1,187.5 ± 183.5	99.0 ± 15.3
Scenario 4	765.4 ± 117.0	63.8 ± 9.8

Note: Scenario 1 denotes patients with CYP2C19*2 and/or *3 variants and taking co-administered CBZ + PHT + PB), Scenario 2 denotes patients with CYP2C19*1*1 genotype and taking co-administered CBZ + PHT + PB, Scenario 3 denotes patients with CYP2C19*2 and/or *3 variants and *NOT* taking co-administered CBZ, PHT, or PB, and Scenario 4 denotes patients with CYP2C19*1*1 genotype and *NOT* taking co-administered CBZ, PHT, or PB.

### 3.5 Performance of the simplified models

**FIGURE 7:**
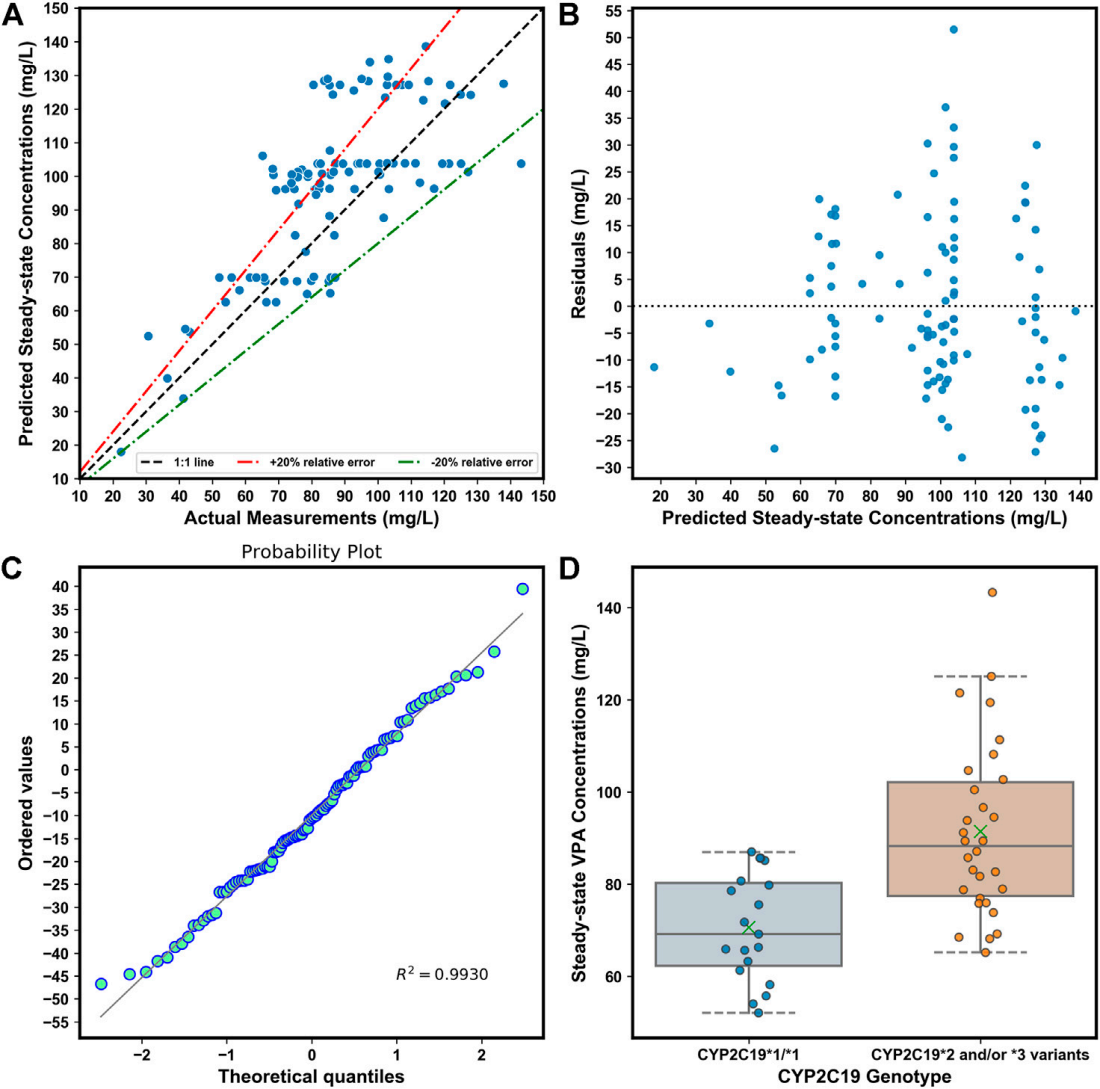
**(A)**. Comparison of predicted and observed $C_{ss}$ values of VPA on the independent external dataset based on the simplified optimum XGBoost model. The range between red and green dotted lines represents +20∼-20% relative errors, i.e., the predicted values within ±20% of the observed values. **(B)** Residuals plot of residuals *versus* the predicted $C_{ss}$ values. **(C)** Probability plot of the residuals. **(D)** Comparison of observed $C_{ss}$ of VPA between patients with CYP2C19*1*1 genotype and CYP2C19*2 and/or *3 variants at a dosage regimen of 500 mg/bid on our independent external dataset. The green multiplication sign indicates the mean $C_{ss}$ values of VPA.

**FIGURE 8:**
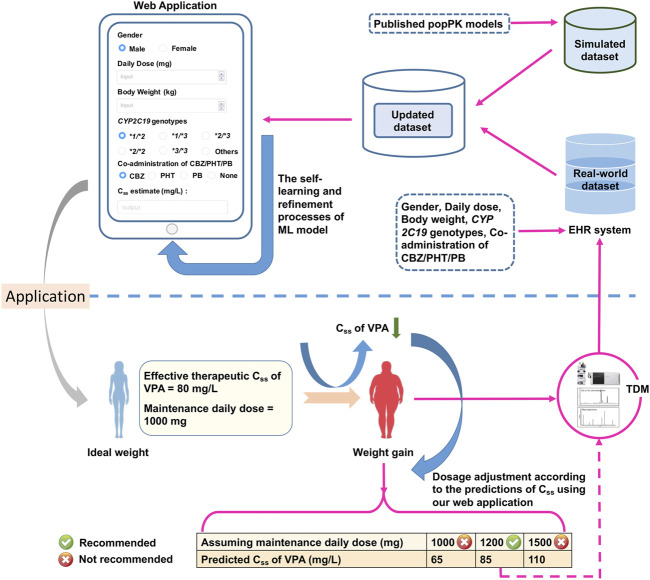
The designed web application for real-time estimations of the value of $C_{ss}$ of VPA based on the simplified optimum XGBoost model. The database can be updated by integrating our simulated dataset with empirical data that are automatically crawled from the electronic health record (EHR) system. This may enhance the self-learning and refinement of the ML model.

The simplified XGBoost model by omitting the features of Single Dose, ALB, t , and τ , yielded reduced performance on the validation cohort, with an MAE of 11.2 mg/L, RMSE of 14.7 mg/L, MRE of 5%, and IR of 68.00%, respectively; whereas, its performance has since been upgraded after optimization (Table 6). The simplified optimum XGBoost model also obtained good performance on our independent external dataset (Table 6). About 60.00% of predicted values fell within ±20% of the empirical values (Figure 7A). Figure 7B illustrates no clear patterns of the distribution of the residuals, and Figure 7C shows the residuals were symmetrically distributed, which meets the assumption of normality (*R* ^2^ = 0.9930). In the external dataset (described in Table 4), the mean measured $C_{ss}$ values of VPA in scenarios 3 and 4 were (91.4 ± 18.7) and (70.6 ± 11.3) mg/L, respectively (Figure 7D), which were close to the predicted ${\bar{C}}_{ss}$ of VPA in these scenarios based on the XGBoost model (see Table 7). A snapshot of the workflow of the designed web application based on the simplified optimum XGBoost model is shown in Figure 8.

## 4 Discussion

ML can serve as a bridge between big data and pharmacometrics by providing an efficient computational approach, but the effective utilization of ML tools in pharmacometrics modeling is still in its infancy (McComb et al., 2022). Many attempts have been made to combine ML and pharmacometrics to advance MIDP, such as the fast screening of covariates in popPK models using ML. However, the ML-based integration of covariates in different popPK models, to our knowledge, is another potentially interesting but unexplored application of ML in pharmacometrics.

In this work, we have first proposed an innovative approach to integrate covariates in multiple previously published popPK models of VPA in Chinese epileptic patients using MC simulations to construct population-based large datasets for ML modeling. However, several key points need to be addressed before implementation. One is the choice of published popPK models. As mentioned at the beginning of the section “materials and methods,” it is important to select suitable popPK models of VPA due to the differing predictability within models. Another point that involves the size ratio of simulated datasets from different popPK models, is also noteworthy. Due to the potential differences in covariate types in different popPK models, missing values of features are inevitable when merging these simulated datasets from different popPK models to construct the combined dataset for the ML task. These features should usually occur in more than 50% of samples; otherwise, they need to be omitted (Meyer et al., 2018). Hence, it is of crucial importance to determine the partition ratio of different sub-datasets in the combined dataset so as not to remove key covariates. Processing these features with less than 50% missing values usually consists of assigning “Unknown” to categorical variables, or setting them to null for further imputation of the missing values. Furthermore, the proportion of data simulated by using different models, as well as the methods dealing with features with missing values, may have an impact on explaining feature importance and the patterns of influence. For example, an inappropriate proportion of simulated datasets may lead to the learning of an insufficient amount of information on the key factors by ML models. Therefore, the appropriate construction of the combined dataset requires incorporating expert knowledge into the ML modeling process. In this study, we have tried to set the simulated sub-datasets close to the same scale while considering the percentages of missing values of features in the finally generated combined dataset. We also have incorporated our expert knowledge into the construction of the combined dataset and well explained the influence of a predictor in the XGBoost model based on the constructed combined dataset by using explanation methods (e.g., the SHAP analysis). The last point to consider is that, after the data cleaning process including missing data imputation and one-hot encoding, we might have to be concerned about multi-collinearity in features in the finally generated combined dataset before ML modeling because collinearity in the features may affect the performance of ML models. The common method of dealing with this is to remove collinearity from the feature set (Dormann et al., 2013). Nevertheless, the decision regarding whether to retain the features related to each other depends on their interpretation meaning, the severity of multicollinearity, and the performance of XGBoost models.

The ultimate prediction model established with XGBoost achieved a good prediction precision and accuracy in the validation cohort. The prediction behaviors of this “black-box” model were illustrated by SHAP analysis. Our results demonstrated that the daily dosage of VPA was the most important variable. Other variables ranking among the top were as follows: blood sampling time, CYP2C19*2 and/or *3 variants, ALB, BW, single dosage of VPA, and CYP2C19*1/*1 genotype. The SHAP dependent plots indicated the nonlinear relationships between the $C_{ss}$ of VPA and blood sampling time and daily/single dosage of VPA. We intuitively found that the time to peak plasma concentration was 1–2 h in line with previous clinical pharmacokinetics reports of VPA (Gugler and von Unruh, 1980). The positive influence of daily/single dosage of VPA on the $C_{ss}$ of VPA tended to be stable along with increased VPA dose, partly explained by a saturable VPA protein binding status, along with a subsequent increase in unbound VPA associated with increased CL , as VPA is a high protein-binding drug (Lin et al., 2015; Gu et al., 2021). The SHAP plots also showed that the $C_{ss}$ of VPA was positively correlated with ALB and CYP2C19*2 and/or *3 variants, and negatively correlated with BW and CYP2C19*1/*1 genotype, which was generally consistent with the results of our selected popPK models (Lin et al., 2015; Guo et al., 2020). The increased content of ALB in the blood results in less unbound VPA, thereby decreasing the CL . CYP2C19*2 and/or *3 variants are associated with the diminished catalytic activity of CYP2C19. Patients with wild-type alleles for *CYP2C19* are classified as extensive metabolizers associated with lower VPA concentrations, whereas non-extensive metabolizers are those with loss-of-function alleles, resulting in higher VPA exposure (Guo et al., 2020). Regarding the BW, our finding was expected given its association with organ functionality development responsible for drug elimination (Methaneethorn, 2018); this was in accordance with several previous studies that reported an increase in CL and $V_{d}$ with increasing BW (Correa et al., 2008; Methaneethorn, 2017; Xu et al., 2018).

Furthermore, after covariate integration, it was necessary to explore the comprehensive impacts of *CYP2C19* genotypes and co-administered enzyme-inducing antiepileptic drugs on VPA exposure. Our simulations, which were well-verified by our independent external dataset, showed that at the dosage regimen of 500 mg/bid, VPA exposure in patients with CYP2C19*2 and/or *3 variants and no co-administered CBZ, PHT, or PB, was approximately 1.74-fold compared to those with CYP2C19*1/*1 genotype and co-administered CBZ + PHT + PB, who would obtain ${\bar{C}}_{ss}$ of (56.9 ± 8.6) mg/L, close to the lower limit of the therapeutic reference range of VPA (50–100 mg/L) recommended by the consensus guidelines for TDM in neuropsychopharmacology (Hiemke et al., 2018). This indicated that in combination with CBZ + PHT + PB, the VPA concentration was decreased in patients with wild-type alleles for *CYP2C19*, which may lead to the risk of ineffective treatment.

We simplified the XGBoost model by omitting several predictors that were infrequently measured during TDM (e.g., ALB), or whose clinical values were inaccurate (e.g., blood sampling time), to develop a clinically easy-to-use model. Compared with the initially proposed XGBoost model, the reduced performance of our simplified XGBoost model indicated the important influences of these features, particularly the blood sampling time and ALB, on the model output. Nevertheless, a 60.00% IR of the simplified optimum XGBoost model on our external dataset suggested its good forecasting performance, considering the prediction accuracy of the predicted TDM within ±30% of the actual TDM in many similar studies that utilized XGBoost models, ranging from 40% to 75% (Huang et al., 2021b; Guo et al., 2021; Zheng et al., 2021; Ma et al., 2022). Based on the simplified optimum XGBoost model, we designed an easy-to-use web application by using only *CYP2C19* genotypes and some noninvasive clinical parameters as an MIPD tool for personalized dosing adjustments. For instance, VPA is known to have both metabolic and endocrinal side effects, and is likely to induce weight gain, which may influence its value of $C_{ss}$ (Corman et al., 1997). Assuming that the effective therapeutic value of $C_{ss}$ of VPA was 80 mg/L under the maintenance of a daily dose of 1,000 mg for a female patient with the ideal BW, adjusted dosing regimens due to weight gain can be recommended by using our web application to reach the target $C_{ss}$ while ignoring the problems of adherence and drug–drug interactions. Furthermore, compared with the static pharmacometrics that requires new models, ML is capable of dynamic learning and retraining (McComb et al., 2022). The database can be updated by integrating our simulated dataset with empirical data automatically crawled from the EHR system. This promotes the self-learning and refinement of the model (see Figure 8).

Despite these promising results, several limitations should be considered. The first was the relatively small sample size of our independent external dataset for performing model validation. In particular, cases of co-administered CBZ/PHT/PB were lacking due to rather few such cases. The second was that some potential key covariates were not included owing to no related published popPK literature. For example, combination with carbapenems can substantially decrease serum VPA concentrations with a mean difference of -43.98 mg/L (Chai et al., 2021), which might cause a huge prediction bias in our model. Future popPK research is needed to evaluate such covariates. The third was that we could not be able to verify whether the covariates from Model-A and Model-B were (partly) correlated or not in the context of pharmacokinetics since they were not identified in the same study. For example, low ALB concentrations have been proved to be associated with weight gain (Basolo et al., 2021), however, the exact relationship between ALB level and BW level remains unclear among Chinese epileptic patients, thus it is difficult to determine which level of ALB corresponds to which level of BW if considering the covariance of the two covariates when creating a virtual population with both covariates. Notably, our ML-based integration approach assumes the covariates derived from different popPK models are not correlated with each other in the context of pharmacokinetic modeling, considering that this ML method generally requires as many candidate influencing factors as possible. The abundant feature information and the massive volume of data can enhance the performance of the ML because it is data intensive. Moreover, the weight of each feature which presents the contribution of a feature to the final prediction can be updated in the ML model’s self-learning and refinement processes by integrating our simulated dataset with the real-world dataset from the EHR system. Finally, as pharmacometrics data are typically limited in size, the methods of model validation in ML are not routinely used in pharmacometrics. There is also a lack of consensus on the relevant definition and approaches (Sherwin et al., 2012; McComb et al., 2022). Nevertheless, a comparison of the predictive performance of the proposed XGBoost model and the two popPK models may be worthy of further examination. Besides, it is difficult to fairly evaluate and quantify the gain of using a combined dataset to develop the XGBoost model compared to a dataset taken from a single popPK model because both the feature dimensions of different datasets and the predictability of different popPK models are different. Whereas, a comparison of the predictive performance of XGBoost models built by using the combined dataset and a dataset derived from a single popPK model may also deserve further research.

## 5 Conclusion

Various popPK models for VPA have been reported; however, covariates affecting pharmacokinetic variability of VPA varied considerably between different popPK models. We innovatively proposed a method to integrate these covariates from multiple previously published popPK models using MC simulations to construct a large combined dataset for ML modeling. Our proposed XGBoost model exhibited excellent performance, the prediction behaviors of which were well-explained by the SHAP analysis. In short, our study highlighted the role of ML, presented as a computational bridge between big data and pharmacometrics, in integrating covariates derived from different popPK models.

## Data Availability

The original contributions presented in the study are included in the article/supplementary material, further inquiries can be directed to the corresponding authors.
